# Association between college health services and contraceptive use among female students at five colleges in Wuhan, China: a cross-sectional study

**DOI:** 10.1186/s12889-016-3612-x

**Published:** 2016-09-05

**Authors:** Lu Long, Zhenhua Chen, Yun Shi, Sheng Wei, Shaofa Nie, Yi Liu

**Affiliations:** 1 West China School of Public Health, Sichuan University, Chengdu, China; 2 Chengdu Municipal Center for Disease Control and Prevention, Sichuan, China; 3 Tongji Medical College, Huazhong University of Science and Technology, Wuhan, China

**Keywords:** Health service, Contraceptive use, Females, College students

## Abstract

**Background:**

College students have a high incidence of unplanned pregnancies in China, which has highly raised public attention. As such, numerous reproductive health services are provided to college students. This study examined whether health services in college lead to contraceptive use among female college students in heterosexual relationships.

**Methods:**

A self-administered questionnaire survey with cross-sectional design was administered among female students in four colleges in Wuhan, China to identify health service factors associated with contraceptive use in the past 6 months.

**Results:**

The analysis revealed that younger female students had lower odds of contraception use, whereas students who reported availability of health-related web sites were more likely to use contraceptives. Female students who reported that contraceptives and birth control counselling were accessible from college health services had greater odds of contraceptive usage. Finally, provision of contraceptives and birth control counselling from school were associated with greater odds of contraceptive use.

**Conclusions:**

Contraceptive-related health services play an important role in reducing unintended pregnancies by directly addressing the contraceptive needs of female students. Programs that provide targeted services may help to reduce high rates of unexpected pregnancies among female students in China.

## Background

In a context of dramatic social change, social and economic development, and the advancement of the digital age, attitudes toward sexuality are becoming much more open, particularly among college students aged 15 to 23 [1]. Research on sexuality and the sexual behavior of Chinese college students over the last decade has shown that, among female students with sexual experience, over 10 % had experienced an unexpected pregnancy [2, 3]. A significant amount of research has been conducted on female involvement in reproductive health decision making, especially for young women [4, 5]. Women’s lives are strongly impacted by pregnancy, and they can improve their sexual health outcomes through the consistent use of contraception to reduce the chances of unintended pregnancy and therefore prevent the need for abortion [5, 6].

The media has become one of the most important sources of reproductive health information among Chinese youth, especially college students [7]. In fact, several studies in China have shown that students’ main source of knowledge on sex and reproduction is the media, including books, magazines and other periodicals, movies, and television [7, 8]. Education from both school and family is the most basic tool to increase young people’s knowledge of and capacity for protecting themselves, but it is largely ignored by teachers and parents because of the cultural norms inhibiting the discussion of sexual matters [9]. A recent study of Internet-based sex education programs has suggested that the Internet is a feasible and effective way of influencing college students’ sexual behavior [9]. In addition, health services, including providing free contraceptives and counseling at college and university student health centers, have been linked to increased contraceptive use and consistency of use among female students [10, 11]. The lack of counseling designed to help improve the results of sexual activity prevents many young people from seeking contraceptives [12–14]. In addition, a recent study suggested that more convenient access to services and contraceptives may be an important intervention to increase contraceptive use [15].

A better understanding of the influences on contraceptive use among female students will help to inform pregnancy prevention efforts. In this study, we expanded on previous research to assess the association between the availability of health services and contraceptive use and consistency of use. We believe that our study will contribute to a better understanding of the kind of reproductive health services that are currently needed and guide interventions and public policies aimed at improving health services and reducing the number of unintended pregnancies.

## Methods

### Study context

This study was conducted in Wuhan Municipality, Hubei Province, from April to June in 2013. The sample for the study was drawn using multistage stratified cluster sampling. The primary sampling units were colleges in comprehensive universities, as defined by the college entrance examination brochures. The five selected colleges (clusters) were of approximately equal size in terms of area and population. In the second stage of sampling, we selected half of the classes in the first to fourth years in every selected college by using simple random sampling. We attempted to recruit all female students in these classes. Only females who had been sexually active in the past 6 months and who responded to a question about contraceptive use were included in the analysis. Ultimately, our analytic sample included 915 female students.

### Data collection instrument, recruitment, and data collection

Students were asked to complete a questionnaire independently in the classroom after a trained staff member provided a brief introduction and instructions. The questionnaire was anonymous and required approximately 10 min to complete. The included questions were developed based on past research and revised using qualitative methods including in-depth interviews with 20 students (four women from each selected college) and five focus group meetings (one at each selected college). The questionnaire was then pilot tested in a group of 50 randomly selected female students in a college. The reliability of the questionnaire was evaluated by comparing the results from two administrations of the same survey to the same 110 female college students with a 2-week interval. More than two-thirds of questions had Kappa statistics over 0.4 (all *p* < 0.05). Cronbach’s alpha coefficient, which was calculated to determine the internal consistency of the scales in the questionnaire, ranged from 0.73 to 0.86. Principal components analysis suggested a good fit and confirmed the internal design of the questionnaire.

Data on contraceptive use were collected through responses to the following statement: “Please check the boxes adjacent to any form of contraception/protection you or your partner are currently using.” A variety of contraceptive options were listed (Table 1). Respondents were given the option of selecting “none” or “other,” in which case they were asked to specify their current method in a text box.

**Table 1:** Distribution of dependent variables and individual, enabling resources, health services and contraception/safer sex options utilized among sexually active female students

Characteristic	Sexually active females	
	n	%
Total	915	100
Fully consistent contraceptive use		
Yes	318	34.7
No	597	65.3
Frequency of contraceptive use		
High	670	73.2
low	245	26.8
Age	15–24^a^	20.5 ± 2.54^b^
Relationship status		
Steady	801	87.5
Casual	114	12.5
Grade		
Senior	534	58.4
Junior	382	41.7
health services^c^		
Provision of health related publications	544	59.5
Availability of designed Web site	633	72.8
school education	254	27.8
Provision of contraceptives	218	23.8
Provision of birth control counseling	135	14.7
Convenient getting contraceptives	118	12.9
Convenient getting birth control counseling	87	9.5
Convenient getting birth control counseling	87	9.5
Contraception/safer sex options^c^		
Condoms	535	61.5
Oral female contraceptives	233	26.8
Rhythm method	217	24.9
Emergency contraception	109	12.5
Contraceptive patches	61	7.5
Spermicides	43	4.9
others	9	1.0

^a^range

^b^mean ± SD

^c^Multiple choice

#### Dependent variables

Our dependent variables were derived from two questions regarding contraceptive use in the past 6 months: “During the past 6 months, have you used contraception to prevent pregnancy or sexually transmitted diseases during most sexual events?” and “How often when you and your partner had sex together did you use contraceptives during the past 6 months?” Using these questions, we constructed two dichotomous dependent variables. The first compared female students who had used contraception in every instance with those who had not used contraception consistently. The second compared students who had frequently used contraception with those who had used contraception with a low frequency.

#### Explanatory variables

We measured three individual characteristics: age, relationship type (steady vs casual), and year in college. A casual partner was one with whom a respondent reported to have had a single encounter. A steady partner was defined as a sexual partner with who a study participant met on a regular basis.

Several developments concerning contraceptive use among college students have occurred in China in recent years, including colleges’ increasing provision of reproductive health publications and development of reproductive health websites for college students [3, 7]. For that reason we wished to assess whether the provision of health-related publications in college and availability of websites were a factor associated with the contraceptive use of college students. The survey assessed whether respondents had access to reproductive health information at their colleges from publications including books, newspapers, websites, and school education. Two response choices (yes and no) were provided for each item. In terms of health services, we measured whether health-related publications (i.e., books and newspapers) and health-related websites were provided in the college and whether health-related courses were offered at the college.

We also measured whether the school provided contraceptives or birth control counseling to female students. Birth control counseling referred to counseling designed to help reduce pressure for college students to become sexually active and improve the results of sexual activity. Additionally, we assessed whether contraceptives and birth control counseling were considered to be convenient to acquire in each college.

### Data analysis and ethical clearance

Data were processed using EpiData version 3.1 and analyzed using Stata version 12.0 software (StataCorp LP, College station, TX: USA). Frequencies and means were calculated for the measured demographic characteristics. Univariate logistic regression models were computed to examine the level of association between individual characteristics, available resources, health services, and contraceptive use. Nominal level independent variables with more than two categories were transformed into dummy variables and assigned reference categories. Variables significant in the univariate analyses (*p* < 0.05) were entered in a multivariate logistic regression model. In the logistic regression model, we adopted the “Enter” method to achieve a final model. The standard for the variable inclusion was based on SLENTRY = 0.05, and the exclusion standard was SLSTAY = 0.10.

The researchers obtained consent from all participants involved in the study. The study was approved by the Research Ethics Committee of Tongji Medical College at Huazhong University of Science and Technology in Wuhan, China. We provided detailed information on the study to the eligible college students and included only those who consented to participate. We obtained written informed consent from all respondents.

## Results

### Sample demographics

**Fig. 1:**
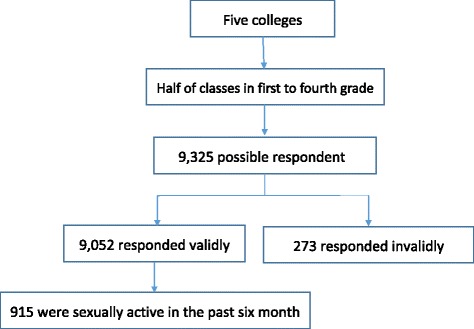
Sampling profile

As is shown in Fig. 1, of the 9325 possible research participants, 9052 responded validly, giving a final response rate of 97.1 %. Women were considered to be sexually active if they reported having had sex with a male partner in the past 6 months. Of the 9052 respondents, 10.1 % (915/9052) of the women reported being sexually active in the past 6 months.

As shown in Table 1, more than one-third of the sexually active women in the sample reported consistent contraceptive use during the recent most recent sexual intercourse event. The overall sample (*N* = 915) of sexually active female college students averaged nearly 21 years of age (mean = 20.5, SD = 2.54). The age range was 15–24. Most of these women had a steady partner (87.5 %). More than two-thirds (72.8 %) of sexually active females reported that health-related websites were provided at their college. Further, about half of the females in the study reported publications on these topics being provided at the college. Approximately 24 % reported that their college provided contraceptives, and 15 % reported that their college offered birth control counseling. Contraceptive acquisition was reported to be convenient by approximately 13 % of the sample, and less than 10 % of respondents reported that birth control counseling was convenient.

### Contraceptive use

There were six types of contraception with more than 1 % usage (Table 1). Condoms were the most popular form of contraceptive, used by 535 respondents. Oral female contraceptives were the second most popular form of contraception.

### Univariate analyses

As is shown in Tables 2, younger students were less likely to report frequent use of contraception compared with older students. Factors associated with the increased likelihood of frequent use of contraception included the availability of health-related websites at the college, the provision of contraceptives at the college and university student health centers, and convenient access to contraceptives and birth control counseling. The students in casual relationships were less likely to be fully consistent with their contraceptive use than were those in steady relationships. Factors associated with the increase likelihood of being fully consistent with contraceptive use included the provision of health-related publications at the college, the provision of birth control counseling at college and university student health centers, and a convenient means of getting contraceptives.

**Table 2:** Associations between health services in school and consistency of contraceptive use among female students

Characteristic	Low vs. High frequency			Not fully vs. Fully		
	Low	High	OR(95 %CI)	Not fully	Fully	OR(95 %CI)
	*N* = 245	*N* = 670		*N* = 597	*N* = 318	
Individual characteristics						
Age						
< 20	40.4	31.2	0.58(0.41,0.83)^*^	36.3	40.7	1.21(0.91,1.61)
≥ 20	59.6	68.8	Reference	63.7	59.3	Reference
Relationship status						
casual	17.4	18.9	0.94(0.72,2.38)	22.2	19.2	0.67(0.44,1.00)^*^
steady	72.6	71.1	Reference	74.6	78.5	Reference
Grade						
Senior	51.4	60.3	1.18(0.86–1.53)	55.2	56.1	1.05(0.82–1.59)
Junior	48.6	39.7	Reference	44.8	43.9	Reference
Provision of health related publications						
Yes	51.4	58.7	1.25(0.86,1.73)	56.9	64.6	1.38(1.04,1.84)^*^
No	48.6	41.3	Reference	43.1	35.4	Reference
Availability of designed Web site						
Yes	59.2	78.1	1.85(1.42,2.56)^**^	72.4	73.5	1.06(0.77,1.45)
No	40.8	21.9	Reference	27.6	26.5	Reference
School education						
Yes	31.3	22.6	0.76(0.54,1.13)	29.0	25.5	0.84(0.61,1.15)
No	68.7	77.4	Reference	71.0	74.5	Reference
Provision of contraceptives						
Yes	10.2	23.1	1.53(1.16, 2.76)^*^	22.5	26.2	1.22(0.88,1.68)
No/don’t know	79.8	76.9	Reference	77.5	73.8	Reference
Provision of birth control counseling						
Yes	11.9	16.6	1.26(0.89,2.10)	12.7	18.5	1.57(1.07,2.30)^*^
No/don’t know	88.1	83.4	Reference	87.3	81.5	Reference
Convenient getting contraceptives						
Yes	12.0	21.6	2.14(1.53,3.28)^***^	9.5	16.3	2.55(1.55,4.20)^***^
No	88.0	78.4	Reference	90.5	83.7	Reference
Convenient getting birth control counseling						
Yes	8.7	16.4	2.34(1.49,3.81)^***^	7.6	10.6	1.43(0.87,2.37)
No	91.3	83.7	Reference	92.4	89.4	Reference

***p < 0 .001; **p < 0.01; *p < 0 .05

### Multivariate analyses

Table 3 displays the multivariate results from the logistic regression analyses. The multivariate analysis revealed that younger female students had lower odds of contraceptive use than did older students, and students who reported the availability of health-related websites were more likely to use contraceptives than were those who reported that no such websites were available. Contraceptive and birth control counseling provision at the college were associated with greater odds of contraceptive use, and those who reported that college health services were convenient for getting contraceptives and birth control counseling also had greater odds of contraceptive use.

**Table 3:** Odds ratios from logistic regression models assessing associations between selected characteristics and contraceptive use

Model 1 (low vs. high)		OR	95 %CI
Age	<20	0.57	(0.43,0.88)*
	≥20	Reference	
Availability of designed Web site	Yes	1.74	(1.26,2.42)**
	No	Reference	
Provision of contraceptives	Yes	1.81	(1.28,2.82)**
	No	Reference	
Convenient getting contraceptives	Yes	1.69	(1.12,2.64)*
	No	Reference	
Convenient getting birth control counseling	Yes	1.91	(1.21,3.15)*
	No	Reference	
Model 2 (Not fully vs. Fully)			
Relationship status	causal	1.42	(0.93,2.21)
	steady	Reference	
Provision of health related publications	Yes	1.64	(1.25,2.37)**
	No	Reference	
Provision of birth control counseling	Yes	1.37	(0.75,1.94)
	No	Reference	
Convenient getting contraceptives	Yes	2.31	(1.45,3.86)**
	No	Reference	

***p < 0 .001; **p < 0.01; *p < 0 .05

In Model 1, which examines high versus low frequency contraceptive use, six variables entered into the final step. In Model 2, which predicts fully consistent contraceptive (vs. not fully consistent), five variables entered into the final step.

#### Model 1 (high frequency vs. low frequency)

We found that students aged under 20 years had a 43 % lower odds of high frequency contraceptive use than that of older students (OR = 0.57, 95 % CI: 0.43–0.88). In contrast, young women who reported that health-related websites were provided at their college had a 74 % higher odds of high frequency contraceptive use than that of students reporting that no websites were provided (OR = 1.74, 95 % CI: 1.26–2.42). Those respondents reporting that contraceptives were provided at college and university student health centers had a 81 % higher odds than that of respondents reporting that they could not access contraceptives this way of using contraceptives with a high frequency(OR = 1.81, 95 % CI: 1.28–2.82). Additionally, young women who reported convenient means for getting contraceptives and birth control counseling had a 69 and 91 % higher odds, respectively, of high frequency contraceptive use (OR = 1.69, 95 % CI: 1.12–2.64 and OR = 1.91, 95 % CI: 1.21–3.15) than those of young women without convenient access to these things.

#### Model 2 (fully consistent vs. not fully consistent)

Young women who reported health-related publications being provided at their college had a 64 % higher odds of fully consistent contraceptive use (OR = 1.64, 95 % CI: 1.25–2.37). In addition, the odds of fully consistent contraceptive use for those who reported a convenient means for acquiring contraceptives was two times greater than these odds for those who did not (OR = 2.31, 95 % CI: 1.45–3.86).

## Discussion

Women can improve their reproductive health outcomes by using contraceptives consistently with sexual partners to prevent unintended pregnancies. A better understanding of factors associated with contraceptive use among female students will help college and family to protect student from unintended pregnancy. Many studies have been conducted on contraceptive use among college students, but few of these have focused on health services in Chinese colleges. This study has extended previous research by assessing the associations between the characteristics of college health services at college and contraceptive use and consistency of use. The study also highlighted the need to design a Chinese reproductive health website focusing on young people and covering important information that young people and adolescents need. Our findings also indicate that better contraceptive-related health services play an important role in reducing unintended pregnancies by directly addressing the contraceptive needs of female students.

However, the findings of this study were limited by several factors. The study sample represents a small proportion of female college students in Wuhan. Individuals who did not want to take part in the research may in fact be more affected than those who did participate. Students with more liberal views on sexuality and sexual practices may be more likely to take and complete a sexuality survey; therefore, our results may not be representative of the general student population. Furthermore, the regularity of contraceptive use was not quantified in this study. A further limitation is that the participating students provided information on contraceptive use retrospectively, whereas contraceptive use would ideally be measured using daily calendars. Finally, no cause–effect relationship could be established because of the cross-sectional nature of the study design. Further research is needed to evaluate contraceptive use and examine in more depth how the factors identified in this study affect contraceptive use.

In our study, we found that certain individual characteristics (including younger age and higher socioeconomic status) are associated with a lower frequency of contraceptive use. Similar to our study, a cross-sectional descriptive study conducted across 49 colleges/universities in seven cities of China also found that, when compared with their classmates aged under 20 years, older students were significantly more likely to use contraception [16]. This may be because older female students have a higher level of knowledge and a greater likelihood of discussing sex and contraception with their parents, peers, and partners than do younger students [3, 17]. However, contraceptive use and consistency has been found to be lower among students older than 35 years [17]. In a univariate analysis, we found that individuals who were in stable sexual relationships were more likely to use contraception in a fully consistent manner, compared with individuals with casual partners, supporting previous studies [18, 19]. However, we did not find any multivariate associations between relationship status and contraceptive use.

We found that the provision of health-related websites by colleges was associated with greater odds of high frequency contraceptive use among female students. This is possibly because the Internet may reinforce the importance of communication between partners about sexual risks and contraceptive use [20]. Previous studies showed that websites were a practical and accessible way of delivering sexual health education to young people, capable of improving behaviors associated with sexual activity, such as the frequency of contraceptive use [12, 21, 22]. Some Chinese reproductive health websites focus on couples with infertility or broadly cover family planning and reproductive health knowledge, but few focus on college students or cover the knowledge and information that college students need [7]. Therefore, better designed websites for sex education should be implemented and popularized among college students. In our study, we found an association between the provision of contraceptives and the odds of contraceptive use, supporting findings from previous research [13]. Possibly because contraceptive costs would account for a non-negligible proportion of daily expenses, women in a poor financial situation were found to be less likely to use contraception consistently. In addition, convenient options for buying contraceptives were associated with a higher frequency of contraceptive use in our study. This supports other studies that have shown that the provision of convenient contraceptive services can improve reproductive health outcomes [23, 24]. However, in China, short-acting contraception suitable for people who have no children is mainly provided without cost to married people by the community health center. Therefore, many unmarried young people—especially college students—cannot access and use reliable and free contraception [16].

We also found that contraceptive counseling among individuals at risk for unintended pregnancy was associated with current contraceptive use among female college students, supporting previous research [25, 26]. School-based contraceptive counselling typically emphasizes unintended pregnancy, sexually-transmitted infections, and other potential risks associated with sex. This type of counseling has a particularly important role in promoting safe sex practices and in preventing multiple unplanned pregnancies and repeat abortions among college students. Our findings indicated that contraceptive counseling may be an important intervention to increase contraceptive use. Additionally, providing free contraceptives at college and university student health centers is associated with increased odds of contraceptive use and consistency of use. China has a strong network of family planning services, with many types of contraceptives provided free of charge. However, family planning services specifically targeting unmarried college students are still absent. Only a few colleges are able to provide free contraceptive services to their students. Efforts should be made to improve contraceptive counseling and services, which would increase the effective use of modern contraceptives [27]. Incorporating this idea into the package of contraceptive services provided at student health centers in colleges could benefit students, and these strategies may help to improve their access to contraceptives [28, 29].

## Conclusions

To prevent negative sexual health outcomes in the college student population, the government must note the importance of sex education among college students, and program efforts must work to instill safe sexual behaviors in this population. Our findings highlighted the need to design a Chinese reproductive health website focusing on young people or covering important information that young people and adolescents need, and to guide young people to visit such a website. Additionally, contraception and birth control counseling are important intervention tools that increase the use of contraception. Better availability of contraceptives plays an important role in reducing the number of unintended pregnancies by directly addressing the contraceptive needs of female students. It is our hope that our findings will guide further interventions and public policy to improve contraceptive availability and reduce unintended pregnancies.
